# Correlation Between *In Vivo* Reflectance Confocal Microscopy and Horizontal Histopathology in Skin Cancer: A Review

**DOI:** 10.3389/fonc.2021.653140

**Published:** 2021-03-08

**Authors:** Giuseppe Broggi, Anna Elisa Verzì, Rosario Caltabiano, Giuseppe Micali, Francesco Lacarrubba

**Affiliations:** ^1^ Department of Medical, Surgical Sciences and Advanced Technologies “G.F. Ingrassia”, Anatomic Pathology, University of Catania, Catania, Italy; ^2^ Dermatology Clinic, University of Catania, Catania, Italy

**Keywords:** horizontal histopathology, reflectance confocal microscopy, skin cancer, correlation, horizontal histopathological sections

## Abstract

In dermatopathological daily practice, vertical histopathology sections are classically used to analyze skin biopsies. Conversely, horizontal histopathological sections are currently used for the diagnosis of some types of alopecia. In the last years the morphological findings obtained by horizontal histopathology have been correlated to those obtained by *in vivo* reflectance confocal microscopy which provides the same “point of view” of the skin. This review paper emphasizes the strong matching and correlation between reflectance confocal microscopy images and horizontal histopathology in cutaneous neoplasms, further demonstrating the strong reliability of this innovative, non-invasive technique in the management of skin tumors.

## Introduction

One of the major application fields of dermatological research has always been the identification of new diagnostic tools capable of improving the diagnostic precocity and accuracy of skin neoplasms (1, 2). In the last decade, *in vivo* reflectance confocal microscopy (RCM) is gradually establishing itself as a non-invasive diagnostic technique for several skin diseases, being able to provide a horizontal high-resolution “point of view” of the skin, from the stratum corneum to the papillary dermis; horizontal skin images up to a 250 μm of maximum depth may be studied through this technique (3–6). The use of RCM in the diagnostic approach to many inflammatory and neoplastic skin diseases is still increasing, representing one of the major diagnostic aids in the dermatological clinical practice (7). However, the horizontal “point of view” provided by RCM does not allow an optimal correlation with classical histopathology that, as known, produces a full-thickness vertical overview of the skin (8, 9). Instead, horizontal histological sections (HHSs) allow a better correlation as they reflect the same skin plane observed by RCM (10).

The possibility of optimally comparing horizontal histopathology and RCM images represents a relatively new trend, and quite a few papers have been published in this field regarding both inflammatory and neoplastic disorders (11–17). The purpose of this review paper is to establish the “state of the art” on RCM and HHS findings in skin tumors, emphasizing how well horizontal histopathology reflects the images provided by RCM.

## Squamous Cell Carcinoma *in Situ* (Bowen’s Disease)

**Figure 1:**
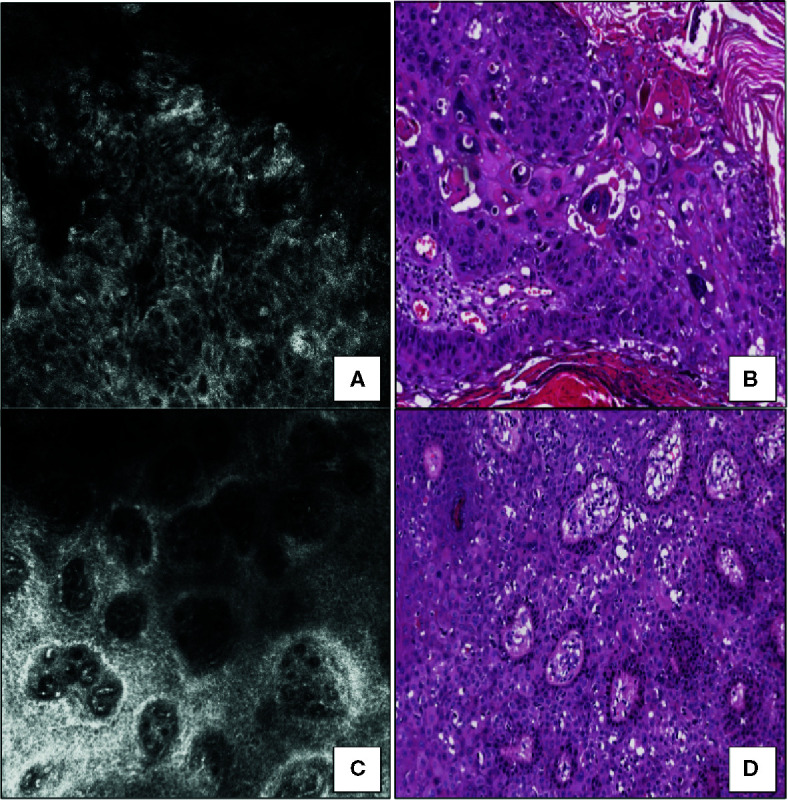
Squamous cell carcinoma *in situ*. **(A)** RCM image at the stratum spinosum showing a marked loss of the normal honeycomb pattern (architectural disarray) due to the presence of markedly variable size, shape, and nuclear morphology keratinocytes. **(B)** Horizontal histopathology at the same level revealing neoplastic keratinocytes with high-grade nuclear atypia (hematoxylin and eosin; original magnification 400×). **(C)** RCM image at the dermoepidermal junction showing dilated blood vessels within enlarged edged dermal papillae. **(D)** Horizontal histopathology at the same level confirming the RCM finding (hematoxylin and eosin; original magnification 100×).

Squamous cell carcinoma *in situ* (SCCis) represents the earliest and non-invasive form of squamous cell carcinoma, in which, by definition, the neoplastic cells do not infiltrate the basement membrane and therefore lack distant metastatic potential (14). SCCis mainly affects photoexposed skin of elderly, and the head and neck are the most commonly affected sites (14). Clinically, SCCis arises in the form of flat/raised, reddish/brownish in color, often scaly, papules or plaques; due to the low specificity of the clinical presentation, further non-invasive diagnostic tools, such as dermoscopy and RCM, are often required to enhance the diagnostic accuracy of SCCis (14, 18). The detection of “red dots”, representing glomerular vessels in the superficial dermis, is the most typical dermoscopic finding of SCCis (18). In addition, RCM has been also validated as useful diagnostic tool and its application in the dermatological practice has been supported by the perfect matching with HHS found by our research group (14). SCCis shows the following RCM features (14) (**Figures 1A, C**): i) at the level of stratum corneum, highly refractive amorphous structures and sporadically polygonal, nucleated cells; ii) at the level of the stratum granulosum/spinosum, marked architectural disarray, consisting of keratinocytes highly variable in size, shape, and nuclear morphology; scattered bright dendritic cells may also be found; iii) at the level of the dermoepidermal junction, large rounded dark areas, corresponding to enlarged dermal papillae. Horizontal histopathology perfectly matches with the previous reported RCM findings (14) (**Figures 1B, D**): hyperkeratosis and parakeratosis are the histopathological causes of the refractive amorphous structures and the nucleated cells observed in the stratum corneum at RCM; the loss of architectural array visible in the stratum granulosum/spinosum at RCM reflects the presence of atypical keratinocytes with nuclei of variable size and shape along the entire thickness of epidermis; some S-100 positive, CD1a negative and Melan-A negative dendritic cells may be occasionally found scattered among the neoplastic cells; lastly, at the dermoepidermal junction, HHSs show enlarged dermal papillae containing glomeruloid capillary vessels, corresponding both to the rounded dark areas and to the “red dots” observed at RCM and dermoscopy, respectively. Since the horizontal histopathology does not allow to evaluate the possible presence of dermal invasion, the concept that its use is only for the purpose of comparing it with the RCM findings, in order to further validate the diagnostic use of RCM, must be emphasized.

## Mycosis Fungoides With Patch Lesions

**Figure 2:**
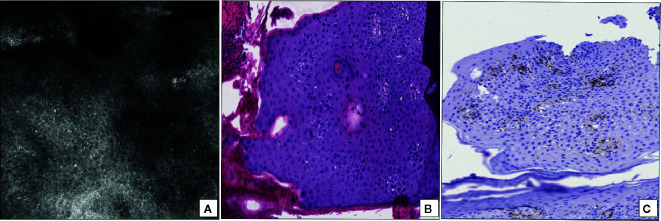
Mycosis fungoides with patch lesions. **(A)** RCM at the stratum spinosum revealing a diffuse epidermal disarray with scattered small hyperreflective cells (epidermotropic lymphocytes). **(B)** Horizontal histopathology at the same level showing the presence of lymphocyte epidermotropism (hematoxylin and eosin; original magnification 400×). **(C)** Immunohistochemical staining for CD4 revealing the CD4-positive phenotype of epidermotropic T-lymphocytes (immunoperoxidase staining; original magnification 350×).

Mycosis fungoides (MF) is the most frequent T-cell lymphoma of the skin and seems pathogenetically related to a monoclonal T-cell receptor (TCR) gene rearrangement, leading to a monoclonal proliferation of cutaneous CD4-positive T lymphocytes (19, 20). Clinically, MF exhibits a higher predilection for dark skin (2:1) males (2:1) and, in its classical form, presents a slow-growing clinical course with a progressive shift from patches to plaques and, in final stages, tumors (19, 20). A variable combination of patches, plaques and tumors is frequently observed in MF with tumor lesions (20). Both clinical presentation and histopathology of MF are often non-specific, especially when it occurs in the form of patchy lesions, to such an extent that multiple biopsies are often necessary to obtain a definitive diagnosis (19, 21). RCM may improve the diagnostic accuracy of MF (13, 22, 23). In the upper portion of epidermis, epidermal disarray with disruption of the normal “honeycomb” appearance and sometimes hyporefractive areas, combined to the detection of small sized bright cells interspersed within epidermal layers are usually identifiable with RCM (13) (**Figure 2A**); the same bright cells are found at the dermoepidermal junction both inside and around dermal papillae, visible as round darker areas (13). RCM features of MF perfectly match with HHS (13): the presence of spongiosis, epidermotropic CD4-positive lymphocytes (**Figures 2B, C**) forming Pautrier’s microabscesses and band-like distributed CD4-positive lymphocytes at dermoepidermal junction are the histopathological “mirror” of what is detectable with RCM. In addition, the differential diagnosis with eczematous disorders can become more straightforward using RCM (13), that shows in the stratum spinosum widespread round, deeply dark areas, intercellular spaces and few mildly bright cells: these findings are confirmed by horizontal histopathology, displaying marked spongiotic features combined to a less conspicuous lymphocytic exocytosis than MF (13).

## Eccrine Poroma

Eccrine poroma (EP) is a sweat gland derived adnexal tumor, first described by Pinkus in 1956 (24), that clinically arises as a slow-growing, sometimes ulcerated, reddish, and firm in consistency nodule, mostly located to the acral regions (25, 26). Usually, EP has a benign clinical course, even if a malignant counterpart, called “porocarcinoma” and characterized by low distant metastatic potential, has been also described (27). EP usually occurs on photodamaged skin, mimicking cutaneous malignancies, such as basal cell carcinoma (BCC), squamous cell carcinoma (SCC) or malignant melanoma (MM) (25, 26). Although the definitive diagnosis of EP is still based on conventional histopathology, non-invasive techniques, including dermoscopy and RCM, allow ruling out malignant conditions, and to suspect a benign adnexal neoplasm (28, 29). Dermoscopically, EP usually presents milky red areas at the periphery of the lesion and a polymorphous vascular pattern in the center, including glomerular, flower-like and dotted vessels (30). RCM shows a uniformly well-circumscribed neoplasm, consisting of hyper-reflective clusters surrounded by a darker stromal component (28, 30). Neoplastic cells are bright and homogeneous in size and shape, with round and dark nuclei, and may be arranged around non-reflective rounded areas (28, 30). Deeper sections show a richly vascularized stroma intermingled with tumor nests (28, 30). RCM images of EP correspond well with HHS (28, 30): neoplastic cells are monomorphic, cuboid-shaped, arranged in basaloid nests and occasionally forming round/slit-like ducts with eosinophilic material inside; these ducts strongly match with the non-reflective round dark areas visible with RCM and represent foci of ductal differentiation of EP. Bright uniformly shaped and sized cells interspersed within the tumor island or scattered in the upper dermis are often present at RCM in the pigmented variant of EP (28); these cells histologically correspond to melanocytes and melanophages, respectively. The presence of melanocytes in pigmented EP makes the differential diagnosis with MM mandatory: neoplastic melanocytes in MM are usually more irregularly shaped/denditric or fusiform than those observed in pigmented EP (31, 32).

## Disseminated Superficial Actinic Porokeratosis

Disseminated superficial actinic porokeratosis (DSAP) represents the most frequent variant of porokeratosis. It clinically presents as multiple scaly macules with a whitish central area surrounded by a slightly raised rim that mainly occurs on photoexposed regions (33). Dermoscopy frequently shows a double free edged scaly rim, whitish in color, representing the dermoscopic equivalent of the cornoid lamella, that is the histopathological hallmark of porokeratosis (34, 35). RCM may be useful in the diagnostic approach to DSAP, and its finding has been validated on the basis of the correlation with HHS (36). At RCM, architectural disarray with loss of the normal “honeycomb” pattern is observed in the center of the lesion (36); proceeding towards the periphery, a less refractile destructured area, containing more refractile amorphous substance (cornoid lamella) and surrounded by normal skin with regular “honeycomb” array, is found (36). HHS strongly matches with these RCM features and shows columns of parakeratosis (cornoid lamella) combined with moderately atypical keratinocytes (36).

## Solitary Mastocytoma

**Figure 3:**
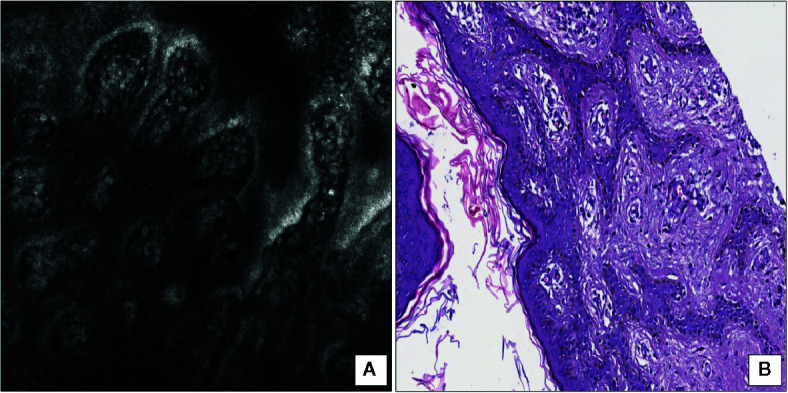
Solitary mastocytoma. **(A)** RCM at the level of dermoepidermal junction showing multiple, large and rounded bright cells within dilated dermal papillae. **(B)** Horizontal histopathology at the same level revealing the presence of round mastocytes with pale and granular cytoplasm within dermal papillae (hematoxylin and eosin; original magnification 150×).

The term “mastocytosis” includes a wide spectrum of diseases caused by a clonal proliferation of mast cell and affecting simultaneously or at different times several organs, including the skin, bone marrow, liver, spleen, and lymphatic system (37). Based on the involved organs, the World Health Organization identifies two different variants of mastocytosis: cutaneous mastocytosis, if the disease exclusively affects the skin, and systemic mastocytosis, if there are other organs affected, regardless of the skin. Furthermore, cutaneous mastocytosis may be clinically further subdivided into maculo-papular cutaneous mastocytosis, diffuse cutaneous mastocytosis, and cutaneous mastocytoma (38). The latter includes not only the cases when there is a single cutaneous lesion (solitary mastocytoma; SM), but also those in which up to three skin lesions are seen (38). Clinical presentation of SM is variable and ranges from brownish/reddish macules to papules, plaques and nodules, showing swelling spontaneously or after rubbing (Darier’s sign). Zhang et al. (39) first described RCM findings of mastocytosis in a huge group of 200 patients, including all different clinical presentation; regardless of the specific variant examined; all cases showed similar RCM features: the absence of aggregates of bright element in the context of finely granular and edematous papillary dermis was a constant finding. Following these results, our group first described more specific RCM features of SM and correlated them with HSS for validation (15): in particular, the presence of enlarged dermal papillae, containing tortuous vessels and large, uniformly round-shaped, bright cells at the level of dermoepidermal junction (**Figure 3A**) perfectly matched with the finding of aggregates of round, CD117-positive mastocytes with granular cytoplasm located to dermal papillae on HHS (**Figure 3B**).

## Melanocytic Tumors

While the introduction of dermoscopy has definitely represented a turning point in the diagnostic accuracy of melanocytic tumors, allowing the detection of some architectural patterns corresponding to specific histopathological features, in recent years RCM has emerged as a valid tool capable of providing architectural and morphological information at the cellular level (40–42); in particular, the combined use of dermoscopy and RCM proved to increase the accuracy for facial tumor detection, compared with RCM alone (43).

Braga et al. (17) compared RCM findings of melanocytic tumors and HHS. They selected four MMs and two benign nevi and compared specific dermoscopic patterns of cutaneous MM such as pigment network, irregular globules and pseudopods, and their benign counterparts, detectable in nevi, to RCM findings and both vertical and horizontal histopathology. Regarding the pigment network, two melanomas showed two different types of atypical network: the first MM presented on RCM a proliferation of bright dendritic cells at the level of dermoepidermal junction, some of them protruding from the epidermis to the superficial dermis to form “bridges”; conventional vertical histopathology revealed an *in situ* melanoma, and HHS showed the same features observed on RCM, confirming the presence of many atypical Melan-A-positive melanocytes surrounding dermal papillae and bulging into dermis. RCM of the second MM with an atypical pigmented network showed at dermoepidermal junction atypical nests of both rounded and elongated hyperreflective melanocytes combined to an architectural disarray of dermal papillae and some bright cells or small dots within dermal papillae; vertical histopathology revealed an *in situ* melanoma, and RCM findings were confirmed by HHS showing pleomorphic melanocytes arranged in nests and presence of lymphocytes within dermal papillae. Based of RCM, Braga et al. (17) were also able to discriminate dermoscopic globules in nevi and melanomas on the basis of morphological atypia: both RCM and HHS showed small nests of monomorphous non-atypical bright melanocytes non-connected with epithelium in nevi and larger nests of pleomorphic neoplastic melanocytes in MMs. Lastly, pseudopods were not characterized by morphological atypia on RCM, corresponding to peripherally visible, confluent clusters of pigmented neoplastic melanocytes on horizontal histopathology. Navarrete-Dechent et al. (16) also matched the dermoscopic sign “circle within a circle” of lentigo maligna (presence of pigmentation within and around hair follicles) with its RCM and HHS: RCM revealed the presence of hair follicles surrounded by numerous dendritic bright melanocytes and layers of keratinocytes filled at the periphery with rounded/elongated hyperreflective melanocytes. HHS strongly overlapped with RCM, showing a high pigmentation of the keratinocytes of the basal layer of the epidermis combined with an increased number of junctional melanocytes.

As previously mentioned regarding SCCis, also for melanocytic tumors, the use of horizontal histopathology has only the purpose of validating the RCM application in clinical practice without replacing conventional histopathology as diagnostic gold standard.

## Discussion

In dermatology, the majority of skin specimens from biopsy or surgical procedures is analyzed using classical vertical histopathological sections, which represents the diagnostic gold standard. Horizontal histopathology is currently used for the diagnosis of some types of alopecia allowing a more correct visualization of follicular and perifollicular features (44).

More recently, HHS has been used to correlate with the morphological features obtained by RCM which provides the same transversal “point of view” of the skin. In particular, the strong matching and correlation between RCM images and HHS in skin tumors (**Table 1**), as shown in this review, further demonstrates the reliability of this innovative, non-invasive technique in the management of skin tumors. Based on such correlations, some considerations can be made: in SCCis and melanoma RCM may confirm the clinical suspect addressing the correct therapeutic approach; in clinically atypical SM, RCM evaluation may avoid biopsy or excision as it is generally self-resolving; in MF and DSAP, RCM is particularly useful for the selection of the best site for biopsy thus avoiding multiple biopsies often quite bothersome for the patient; a further application of RCM in skin tumors may consist in the early recognition of local recurrences after medical or surgical treatments of the disease (14).

**Table 1:** Correlation between reflectance confocal microscopy and horizontal histopathology in skin tumors: summary.

	Depth	RCM	HHS
**SCCis** (14)	Stratum Corneum	- Hyperrefractive amorphous structures - Polygonal, nucleated cells	- Hyperkeratosis - Parakeratosis
	Stratum granulosum/spinosum	- Architectural disarray - Bright dendritic cells	- Large atypical keratinocytes - Langerhans cells (S-100 +, CD1a+, Melan-A -)
	Dermoepidermal junction	- Enlarged edged papillae with widened dermal papillae - Tortuouscapillary vessels	- Enlarged papillae with widened dermal papillae - Tortuouscapillary vessels
**MF** (13)	Upper epidermis	- Darker spots compared to the surrounding epidermis. - Epidermal disarray and presence of small bright cells	- Spongiosis - CD4-positive T-cellepidermotropism
	Dermoepidermal junction	- Small bright cells scattered within and among roundish hyporefractive areas (dermal papillae)	- CD4-positive lymphocytes infiltrating dermal papillae
**EP** (28, 30)	Epidermis	- Clusters of small, hyperrefractive and uniformly shaped cells with round dark nuclei surrounded by keratin - Parakeratosis	- Monomorphic basophilic neoplastic cells with large and round nuclei surrounded by amorphic keratin - Parakeratosis
	Dermis	- Larger and confluent cell clusters embedded in a denser and highly vascularized stroma- Neoplastic clusters arranged around darker hyporefractive rounded areas- Presence of bright, uniformly shaped and sized cells interspersed within tumor island or scattered in the upper dermis (pigmented variant)	- Increased tumor volume and denser and more vascularized stromal compartment - Intratumoral round or slit-like areas filled with eosinophilic substance (spots of ductal differentiation) - Intratumoral melanocytes or melanophages (pigmented variant)
**DSAP** (36)	Epidermis	- Architectural disarray with loss of the normal “honeycomb” pattern (central zone) - Hyperrefractive amorphous material (cornoid lamella) within hyporefractivedestructured areas, surrounded by skin with regular “honeycomb” pattern (peripheral zone)	- Columns of parakeratosis (cornoid lamella) combined with moderately atypical keratinocytes
**SM** (15)	Dermoepidermal junction	- Tortuous vessels and large, uniformly round-shaped, bright cells within enlarged dermal papillae	- Dermal papillae containing aggregates of round, CD117-positive mastocytes with granular cytoplasm
**MTs** (16, 17)	Dermoepidermal junction	- Atypical pigment network: proliferation of bright dendritic cells, forming “bridge” from epidermis to the superficial dermis (*in situ* melanoma) - Atypical pigment network: atypical nests of rounded and spindled hyperreflective melanocytes combined to an architectural disarray of dermal papillae and some bright cells or small dots within dermal papillae (*in situ* melanoma) - Hair follicles surrounded by multiple dendritic bright melanocytes and layers of keratinocytes filled at the periphery with rounded/elongated hyperreflective melanocytes (lentigo maligna).	- Presence of atypical Melan-A-positive melanocytes surrounding dermal papillae and bulging into dermis (*in situ* melanoma) - Atypical melanocytes arranged in nests and presence of lymphocytes within dermal papillae (*in situ* melanoma) - Heavily pigmented keratinocytes of the basal layer of the epidermis combined with an increased number of junctional melanocytes (lentigo maligna).
	Upperdermis	- Dermoscopic globules: small nests of monomorphous non-atypical bright melanocytes non connected with epithelium in nevi and larger nests of pleomorphic neoplastic melanocytes in melanomas - Non-atypical peripheral pseudopods	- Small nests of non-atypical melanocytes in nevi and larger clusters of atypical neoplastic melanocytes in melanomas - peripheral confluent clusters of pigmented neoplastic melanocytes

RCM, reflectance confocal microscopy; HHS, horizontal histopathological section; SCCis, squamous cell carcinoma in situ; MF, mycosis fungoides; EP, eccrine poroma; DSAP, disseminated superficial actinic porokeratosis; SM, solitary mastocytoma; MTs, melanocytic tumours.

## Author Contributions

All authors listed have made a substantial, direct and intellectual contribution to the work, and approved it for publication.

## Conflict of Interest

The authors declare that the research was conducted in the absence of any commercial or financial relationships that could be construed as a potential conflict of interest.
